# The Mechanistic Understanding of RAD51 Defibrillation: A Critical Step in BRCA2-Mediated DNA Repair by Homologous Recombination

**DOI:** 10.3390/ijms23158338

**Published:** 2022-07-28

**Authors:** Fabrizio Schipani, Marcella Manerba, Roberto Marotta, Laura Poppi, Arianna Gennari, Francesco Rinaldi, Andrea Armirotti, Fulvia Farabegoli, Marinella Roberti, Giuseppina Di Stefano, Walter Rocchia, Stefania Girotto, Nicola Tirelli, Andrea Cavalli

**Affiliations:** 1 Computational and Chemical Biology, Istituto Italiano di Tecnologia, Via Morego 30, 16163 Genoa, Italy; fabrizio.schipani@iit.it (F.S.); marcella.manerba@iit.it (M.M.); francesco.rinaldi@iit.it (F.R.); stefania.girotto@iit.it (S.G.); 2 Electron Microscopy Facility, Istituto Italiano di Tecnologia, Via Morego, 30, 16163 Genoa, Italy; roberto.marotta@iit.it; 3 Department of Pharmacy and Biotechnology, University of Bologna, Via Belmeloro 6, 40126 Bologna, Italy; laura.poppi2@unibo.it (L.P.); fulvia.farabegoli@unibo.it (F.F.); marinella.roberti@unibo.it (M.R.); 4 Laboratory of Polymers and Biomaterials, Istituto Italiano di Tecnologia, Via Morego, 30, 16163 Genoa, Italy; arianna.gennari@iit.it; 5 Analytical Chemistry Lab, Istituto Italiano di Tecnologia, Via Morego, 30, 16163 Genoa, Italy; andrea.armirotti@iit.it; 6 Department of Experimental, Diagnostic and Specialty Medicine, University of Bologna, Via S. Giacomo 14, 40126 Bologna, Italy; giuseppina.distefano@unibo.it; 7 CONCEPT Lab, Istituto Italiano di Tecnologia, Via E. Melen 83, 16152 Genoa, Italy; walter.rocchia@iit.it; 8 School of Health Sciences, University of Manchester, Oxford Road, Manchester M13 9PL, UK

**Keywords:** homologous recombination, DNA repair, chemo/radiosensitizer, synthetic lethality, anticancer drug discovery, PARP, BRCA2

## Abstract

The cytotoxic action of anticancer drugs can be potentiated by inhibiting DNA repair mechanisms. RAD51 is a crucial protein for genomic stability due to its critical role in the homologous recombination (HR) pathway. BRCA2 assists RAD51 fibrillation and defibrillation in the cytoplasm and nucleus and assists its nuclear transport. BRC4 is a peptide derived from the fourth BRC repeat of BRCA2, and it lacks the nuclear localization sequence. Here, we used BRC4 to (i) reverse RAD51 fibrillation; (ii) avoid the nuclear transport of RAD51; and (iii) inhibit HR and enhance the efficacy of chemotherapeutic treatments. Specifically, using static and dynamic light scattering, transmission electron microscopy, and microscale thermophoresis, we show that BRC4 eroded RAD51 fibrils from their termini through a “domino” mechanism and yielded monomeric RAD51 with a cumulative nanomolar affinity. Using cellular assays (BxPC-3, pancreatic cancer), we show that a myristoylated BRC4 (designed for a more efficient cell entry) abolished the formation of nuclear RAD51 foci. The present study provides a molecular description of RAD51 defibrillation, an essential step in BRCA2-mediated homologous recombination and DNA repair.

## 1. Introduction

This study aimed to provide a detailed molecular description of RAD51 defibrillation, a crucial mechanistic step in homologous recombination. This understanding may help researchers to develop molecules to hamper the DNA repair capacity of cancer cells.

Genomic stability is key for cell replication and survival. The biological mechanisms to detect DNA lesions, signal their presence, and promote their repair [1,2] are collectively known as the DNA damage response (DDR) pathway. Misregulation in any DDR component can result in genomic instability, which is conducive to cancer onset and maintenance. For example, rapidly proliferating cancer cells are genetically more unstable than healthy cells, and their survival strongly relies on the DDR, in particular on the upregulation of the error-free homologous recombination (HR) mechanism that repairs highly toxic DNA double-strand breaks (dsDNA) [3]. HR inhibition can be therapeutically beneficial. Increasing DNA instability may directly limit cancer progression [4]. In parallel, it may amplify the effects of chemotherapeutic agents [5], impacting on their efficacy and reducing resistance [6].

RAD51 and BRCA2 are two key HR players and are strongly associated with cancer onset and anticancer therapies. The overexpression of RAD51 has been reported in several cancers and correlates with higher and more efficient HR [7,8]. Moreover, germline mutations of the *BRCA2* gene increase susceptibility to breast, ovarian, pancreatic, and other cancer types [9], which are more sensitive to radio/chemotherapy. Indeed, mutated BRCA2 protein can hamper the proper formation of the BRCA2-RAD51 complex and can lead to radio/chemosensitization [10].

RAD51 is a recombinase of 339 amino acids that is mainly present in an equilibrium between monomers and homo-oligomers in the cytosol in normal cycling cells [11]. Upon DNA damage, RAD51 is recruited from the cytosol and transported into the nucleus, oligomerizing on single-strand DNA (ssDNA) and searching for homologous DNA to repair damaged sequences [12]. RAD51 recruitment and accumulation in the nuclear repairing foci (whose number is often used as a marker of HR functionality [13]) are assisted by BRCA2 [14]. BRCA2 is a large protein (3418 amino acids) that interacts with RAD51 through eight well-conserved 35–40 amino acid sequences known as BRC repeats. It tightly regulates the level of RAD51 subcellular distribution and therefore cells’ HR activity by (i) inducing RAD51 defibrillation both in the cytoplasm to support its nuclear translocation and in the nucleus after DNA repair is completed [15]; (ii) assisting its fibrillation along the damaged DNA, supporting the 3′-5′ directional growth of the fibril [16]; (iii) stabilizing these oligomeric structures needed for recombinase activity, binding them with its C-terminal domain upon its dephosphorylation [11]; and (iv) hiding the RAD51 nuclear export signal (NES), thus allowing its nuclear retention when necessary [15].

Currently, the only estimates of BRCA2-RAD51 binding have been obtained from pull-down assays reporting a nanomolar affinity [17]. Of greater interest are relative comparisons, which report that, of the eight BRC repeats, BRC4 shows the highest affinity for RAD51 [18]. Indeed, BRC4 appears to be critical in controlling RAD51 fibrillation, most likely because it mimics a motif used by RAD51 in its oligomerization. This information comes from the only 3D structure of the RAD51-BRC4 interaction (PBD 1NOW) [19]. The BRC4 repeat seems able to promote RAD51 filament formation via interactions involving an LFDE (Leu-Phe-Asp-Glu) sequence, with a binding that is not expected to perturb the RAD51 interprotomer interface. Conversely, a second binding site in an FXXA (Phe-X-X-Ala) region is positioned at the RAD51 protomer–protomer interface and possesses filament-inhibitory potential [20]. BRC4 is therefore often used as a model to reproduce the BRCA2-RAD51 interaction. However, quantitative data are still infrequent. To our knowledge, just one report provides an apparent Kd of 1–2 µM from an indirect GST pull-down assay [21]. If BRCA2-RAD51 interactions are disrupted in the cytosol, hindering the recruitment of RAD51 monomers by BRCA2, HR is most likely inhibited due to the failure of RAD51 nuclear transport.

Most of the literature, comprising biophysical and structural characterizations, focuses on the interaction of BRCA2 with RAD51 fibrils formed on the DNA to unveil the details of the mechanism of DNA repair. Nonetheless, the BRCA2-RAD51 interaction, as a cancer-related target, can be disrupted directly in the cytosol at the RAD51 recruitment stage. If RAD51 self-assembled fibrils are not recruited as monomers by BRCA2, the HR activity is most likely inhibited due to the failure of RAD51 nuclear internalization. The intrinsic ability of RAD51 to form fibrillar structures (homo-oligomers) in vitro, as well as in the absence of DNA, has already been reported [22,23]. Nonetheless, these RAD51 fibrils, recruited and monomerized by BRCA2 to be internalized in the nucleus as a response to DNA damage, have been poorly characterized and understood.

Here, for the first time, we provide a comprehensive biophysical characterization of the native, self-assembled RAD51 fibrils and their disassembly mechanism in the presence of the BRC4 peptide. The mechanism of action underlying BRC4’s ability to disassemble RAD51 native fibrils is investigated in a concentration-dependent manner utilizing complementary biophysical approaches. A detailed understanding of this molecular mechanism is critical to designing a specific inhibition of RAD51 recruitment at the site of DNA damage. This inhibition should lead to HR failure in cancer cells, which rely on this pathway for survival to a greater extent than normal cells, hence promoting selectivity against cancer. Based on cellular experiments, we propose BRC4 itself as a potential inhibitor of the HR pathway for cancer treatment. Indeed, the peptide can block HR in pancreatic cancer cells while enhancing the effect of chemotherapeutic agents. These data show a proof of concept of BRC4’s ability to act as a chemosensitizer.

## 2. Results

**Figure 1:**
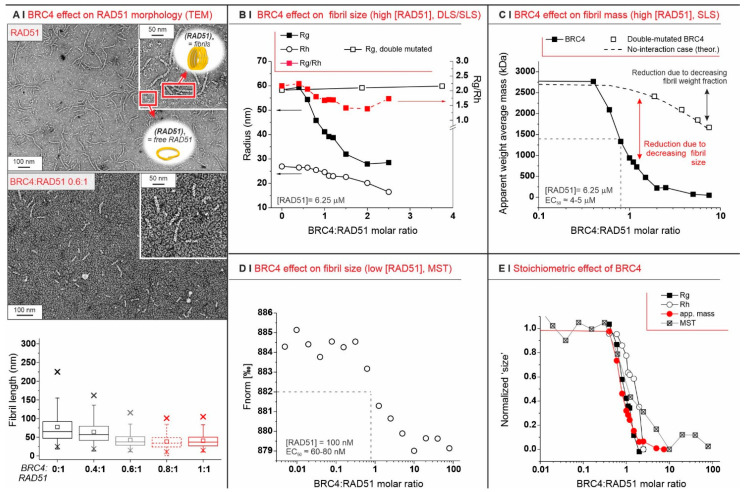
(**A**) Negative staining TEM of 0.1 mg/mL RAD51 fibrils, alone (top) or in the presence of BRC4 (middle, RAD51/BRC4 1:0.6 molar ratio), clearly indicates the latter as an antagonist of RAD51 fibrillation. The fibril length is reported in a box plot as a function of BRC4 concentration (bottom) after fitting TEM data with Gaussian models (see Supporting Information, Figure S3). In the absence of BRC4, most of the fibrils appeared to be 80–200 nm-long, but they may have been fragmented during sample preparation; indeed, static light scattering predicted a higher fibril length (Table S1). (**B**) Evolution of Rg, Rh, and the Rg/Rh ratio (i.e., the aspect ratio) of RAD51 fibrils (6.25 µM = 0.25 mg/mL) upon addition of BRC4 or scBRC4. (**C**) Apparent weight average mass ($\bar{AM_{w}}$ from SLS measurements: it is the average mass of all colloids in the sample) for RAD51/BRC4 suspensions ((RAD51) = 6.25 µM = 0.25 mg/mL) as a function of the peptide/protein ratio. The dashed line was calculated assuming that a compound of the same mass of BRC4 was added and had no interaction with RAD51. The empty squares refer to SLS measurements performed with a double-mutated form of BRC4. (**D**) MST measurements provide an indication of the size (diffusion coefficient) of RAD51 fibrils, which was reduced with increasing RAD51/BRC4 ratios ((RAD51) = 100 nM = 4 µg/mL). (**E**) SLS (Rg and apparent mass data), DLS (Rh data), and MST measurements ($\bar{AM_{w}}$ data) all suggest a stoichiometric (1:1) mode of action for the BRC4-induced RAD51 defibrillation.

In this work, we initially used the BRC4 peptide (residues 1517–1551 of BRCA2), focusing on how it can disassemble the fibrils that RAD51 spontaneously forms (also in the absence of nucleic acids). We expressed the full-length recombinant human RAD51 (N-terminal 6xHis-tagged, see Materials and Methods Section 4.1), a protein of about 40 kDa in its monomeric form, with no DNA contamination (Figure S1). In nondenaturing conditions, however, RAD51, even in the absence of DNA, readily oligomerized and formed aggregates with an elongated, worm-like morphology and a helical organization (Figure 1A, top; Figure S2), as suggested by electron microscopy (EM) data analysis and by the value of the Rg/R_h_ (aspect) ratio calculated from static light scattering (SLS) and dynamic light scattering (DLS) (Table S1). Due to their shape, we will refer to them as RAD51 fibrils.

Depending on their concentration, RAD51 fibrils were differently sized. Zimm plots (Figure S4A) confirm a strong tendency towards aggregation through the negative value of the second virial coefficient A2. Zimm plots also estimate 40 protomers (we refer to RAD51 monomers under nondenaturing conditions as protomers) per aggregate at infinite dilution, which compares to about 70 protomers at a RAD51 concentration of 0.25 mg/mL (6.25 µM; see Table S1 and Figure S4B for the dependency of the radius of gyration Rg on RAD51 concentration, and Figure S4C for the hydrodynamic radius R_h_).

Upon exposure to BRC4, RAD51 fibrils decreased in size. They eventually became invisible in TEM experiments, with large stoichiometric excesses of BRC4 (compare TEM pictures in Figure 1A, top and middle, and see the overall results in Figure 1A, bottom). This was confirmed by size exclusion chromatography (SEC), which indicated that the initial high-molecular-weight fibrils were mostly converted to small-size aggregates and monomers (Figure S5). It was also confirmed more quantitatively using SLS and DLS, which showed a clear decrease in fibril dimensions (Rg (SLS) and R_h_ (DLS)) above a BRC4:RAD51 molar ratio of 0.5 (Figure 1B). Interestingly, the aspect ratio (Rg/R_h_, red squares in Figure 1B) was only marginally affected by the process, indicating that the aggregates maintained an elongated shape, as also seen in the TEM of the residual aggregates. Nevertheless, upon exposure of RAD51 fibrils to a large stoichiometric excess of a double-mutated version of BRC4 (scBRC4), where two amino acids critical for RAD51 binding (F1524, T1526) [24,25] were replaced by alanine residues, the fibrils were still visible, which confirms a direct correlation between RAD51-BRC4 binding and fibril disassembly (Figure S6).

### 2.1. The Defibrillation Is Specific to BRC4

We then used SLS to estimate the overall apparent weight average mass $\bar{AM_{w}}$ of the colloids in a sample, i.e., the linear combination of the masses of all dispersed objects, each weighted by their weight fraction (Appendix A, Equation (A1)). In the absence of the peptide, RAD51 fibrils had a weight fraction of 1; thus, $\bar{AM_{w}}$ was the actual mass of the fibrils. With a compound unable to interact with the fibrils,$\;\bar{AM_{w}}$ would slowly decrease (see dashed line in Figure 1C) because the weight fraction of the fibrils (not their size) decreased. When using the double-mutated scBRC4 peptide, $\bar{AM_{w}}$ followed the theoretical “no-interaction” prediction even with very significant stoichiometric excesses (empty squares in Figure 1C).

Defibrillation is a stoichiometric process independent of RAD51 concentration. Since RAD51 fibrillation depends on concentration, we studied defibrillation using microscale thermophoresis (MST). This allowed us to use RAD51 at a considerably lower concentration than SLS: 100 nM (4 µg/mL) vs. 6.25 µM in SLS experiments. Here, too, however, the process peaked around the BRC4/RAD51 stoichiometric equivalence (Figure 1D). Moreover, MST data closely mapped those from SLS (Rg and $\bar{AM_{w}}$) and DLS (R_h_), with all techniques indicating that BRC4 defibrillation is a strictly stoichiometric process (Figure 1E). When using the double-mutated scBRC4 peptide, MST also showed no binding of the peptide to RAD51 (data not shown).

**Figure 2:**
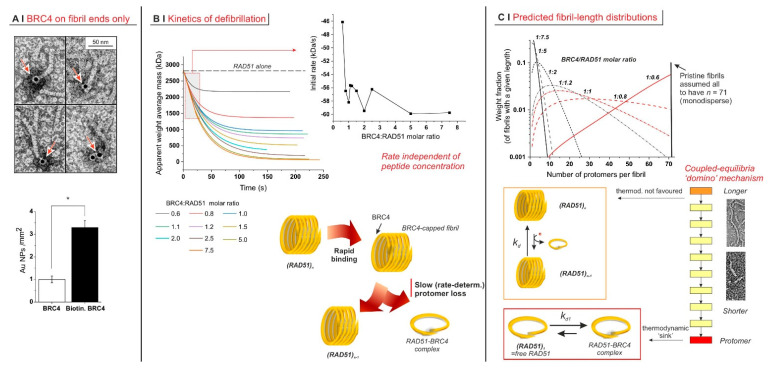
(**A**) Localization of biotinylated BRC4 peptide by avidin–gold labelling. The histogram below shows the different densities of gold nanoparticles when nonlabelled BRC4 or biotinylated BRC4 was used. The asterisk indicates a statistically significant difference (two-tailed Student’s *t*-test * *p* < 0.01). The labelled peptide was predominantly localized at only one of the fibril termini (23%, n = 67). In the remaining cases, it appeared to be bound to isolated RAD51 monomers (73%, n = 67). In the pictorial representation of RAD51 (below), its protomer was assumed to adopt a helical morphology, as evidenced by TEM images, and the fibrils were assumed to comprise aligned helices. (**B**) $\bar{AM_{w}}$ recorded as a function of time on RAD51/BRC4 suspensions ([RAD51] = 6.25 µM = 0.25 mg/mL) with various peptide/protein ratios. The data were fitted with an exponential function. The initial decrease rate, recorded as a function of the BRC4/RAD51 molar ratio (inset), was obtained as the derivative of the function at time zero. (**C**) Simulated weight distribution of RAD51 fibrils with different lengths was produced upon addition of variable amounts of BRC4. The model used is described in Appendix A and was fed with $\bar{AM_{w}}$ data. Mechanistically, the whole process was based on a succession of losses of a RAD51 unit. Each individual step appeared to be characterized by a rather unfavourable constant. However, their coupling and above all the presence of a final “sink” drove the process to completion. Indeed, the complexation of RAD51 with BRC4 appeared to be thermodynamically favoured, with a dissociation constant kd1 calculated by our model estimated to be mostly in the range 0.1–1 µM.

Defibrillation may proceed gradually from a fibril terminus, instead of being based on BRC4 attacking fibrils at random locations. Upon incubating fibrils with biotinylated BRC4, virtually all particles bound to fibrils were localized at only one of their termini (Figure 2A). Notably, around 75% of the particles were bound to isolated RAD51 units, which must therefore be considered to be RAD51/BRC4 complexes.

Further, when monitoring defibrillation kinetics via SLS (Figure 2B, top left graph), we noticed that the rate of the process was largely independent of the amount of BRC4 (Figure 2B, top-right graph, RAD51 concentration constant).

Notably, EC_50_ values were markedly different at different concentrations (compare Figure 1B,D). This indicates that the concentration of the limiting component (i.e., (RAD51)) was greater than or equal to the apparent dissociation constant Kd of the process and that the experiments were run under the titration regime (as opposed to the binding regime, which allows one to approximate Kd with EC_50_ [26]). It is, therefore, reasonable to assume that Kd was less than the lowest EC_50_ value recorded, i.e., ≤60 nM. Defibrillation, however, is not a single equilibrium but rather a succession of coupled steps, which can each be considered with their dissociation constant *k_d_*. We used a mathematical model (see Appendix A) for their semiquantitative estimation from $\bar{AM_{w}}$ data, assuming (i) independence of *k_d_* on fibril length; i.e., we assumed all steps had the same dissociation constant and (ii) monodispersity of original fibrils; i.e., at the beginning of the process, we ignored the fibril size distribution. Our simulation clearly showed a decrease in fibril length (number of protomers per fibril) with an increasing BRC4/RAD51 molar ratio (Figure 2C, top). The products of intermediate defibrillation steps had a large dispersity in size, which is a common feature of all step-based processes, such as step-growth polymerization.

The *k_d_* calculated for all BRC4/RAD51 molar ratios was essentially constant, with a dimensionless value of 33.7 ± 0.5. This suggests that the intermediate steps may not be thermodynamically favoured, whereas the last event (the complexation of free protomer) may act as the thermodynamic “sink” driving the “domino” chain of coupled steps (Figure 2C, bottom).

### 2.2. BRC4 Can Inhibit HR Activity

**Figure 3:**
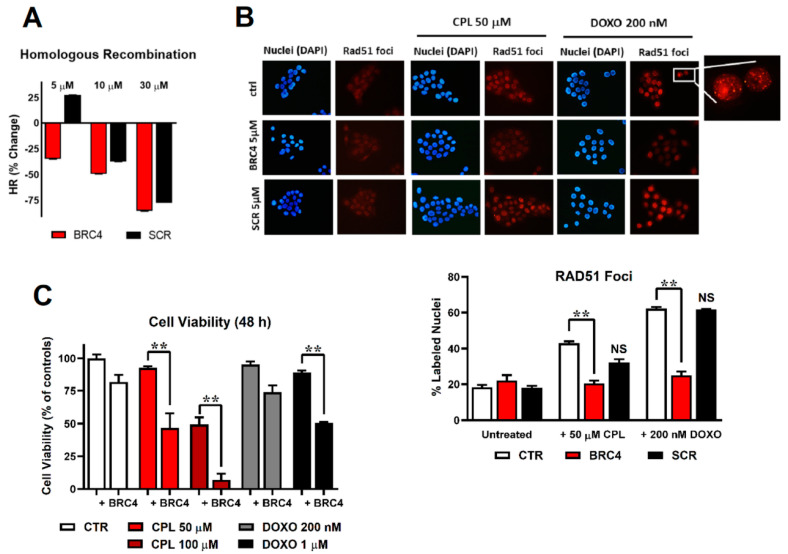
(**A**) homologous recombination (HR) assay: evaluation of the percentage of HR inhibition at 5, 10, and 30 μM BRC4myr peptide (red) or scrambled myristoylated peptide scBRC4myr (black). (**B**) Immunofluorescence detection of RAD51 foci in cell nuclei. BxPC-3 cells were exposed to 50 μM cisplatin (CPL) or 200 nM doxorubicin (DOXO) alone or in combination with 5 μM BRC4myr or scBRC4myr peptides. Representative pictures showing DAPI-stained cell nuclei and the corresponding immune labelling of RAD51 localization. RAD51 labelling appeared mainly in CPL- and DOXO-exposed cells. A higher magnification detail of the 200 nM DOXO-treated sample is included. The bar graph shows the percentage of RAD51-lableled nuclei estimated by analyzing approximately 300 cells for each treatment. Data were statistically evaluated by applying the two-way ANOVA with multiple comparisons. A statistically significant difference was found between cells treated with BRC4myr in combination with CPL or DOXO compared to cells treated with CPL or DOXO alone. (**C**) Cell viability assessed in BxPC-3 cultures exposed to CPL (50 or 100 µM, 1.5 h) or doxorubicin (DOXO, 200 nM or 1 µM, 1.5 h) and treated for 48 h with 5 µM BRC4myr. During the experiment, cultures were maintained in a medium without serum supplementation to avoid interference of serum with peptide effects (degradation by proteases). Data were analyzed by two-way ANOVA with multiple comparisons. BRC4myr significantly increased the effects of both CPL and DOXO with *p* < 0.01 (**).

We then used BxPC-3, a pancreatic adenocarcinoma cell line expressing a functional BRCA2 [27]. In its native form, BRC4 was unable to impair HR (Materials and Methods, Section 4.3) due to inefficient cytoplasmic penetration. However, its myristoylated version (BRC4myr: a reducible disulfide links a C-terminal BRC4 Cys with a myristoylated Cys-Lys(Myr)-Lys-Lys-Lys, allowing both membrane permeation and intracellular release) showed a dose-dependent HR inhibition of up to 85.1% at 30 µM with an estimated IC_50_ ≈ 10 µM (Figure 3A). The localization of RAD51 in BxPC-3 nuclei provided additional evidence for a compromised HR. The large number of nuclear RAD51 foci induced by exposure to 50 µM cisplatin (CPL) or 200 nM doxorubicin (DOXO) virtually disappeared in the presence of 5 μM BRC4myr (Figure 3B). Similar inhibitory HR activity of exogenous BRC4 expression has already been reported in MCF-7 cancer breast cell lines [28].

### 2.3. HR Impairment Can Potentiate the Cytotoxic Effects of Anticancer Drugs

Treatment of BxPC-3 cells with the myristoylated double-mutated scBRC4 peptide (scBRC4myr) showed no HR inhibition at 5 µM peptide concentration only. At higher concentrations, HR inhibition was observed, although this was weaker than for BRC4 (Figure 3A). It may be due to a weak RAD51-scBRC4 interaction supported by the presence of cofactors and interactors lacking in in vitro experiments or off-target bindings. The localization of RAD51 in BxPC-3 nuclei upon exposure to 50 µM cisplatin (CPL) or 200 nM doxorubicin (DOXO) was also weakly affected (no statistically significant difference) by the presence of 5 μM scBRC4myr compared to the effect induced by BRC4, proving the existence of a direct correlation between RAD51-BRC4 binding and HR inhibition (Figure 3B). When BxPC-3 cultures were pre-incubated with 5 µM BRC4myr and maintained in its presence during CPL or DOXO treatment, the toxicity of the chemotherapeutic agents was significantly increased (Figure 3C).

## 3. Discussion

We have shown here that, in the absence of DNA, recombinant RAD51 had an intrinsic tendency to form worm-like fibrils with a helical organization and dimensions that depend on protein concentration. It seems reasonable to hypothesize that this dependence is caused by an equilibrium of terminal association between RAD51 protomers and fibril termini. We are inclined to exclude the idea that higher concentrations increase the nucleation of new fibrils. This is because it would cause a fraction of smaller fibrils at higher RAD51 concentrations (as happens for fibrin [29]). Upon BRC4-RAD51 binding, the peptide reverts the fibrillar self-association of RAD51.

Mechanistically, our results suggest that during cytosolic RAD51 recruitment, BRCA2 (here mimicked by BRC4) may disassemble fibrils gradually from their termini rather than through attacks at random positions along the fibril. All indicators of fibril size (mass, dimension, diffusion coefficient) decreased very gradually, with the midpoint of the process being observed to be close to the 1:1 BRC4/RAD51 stoichiometry. Further, TEM, SLS, and DLS have shown that the fibrils preserved an elongated morphology (a high-aspect ratio) during the process, and BRC4 localized at the fibril termini. Finally, if defibrillation proceeded from fibril termini, the number of fibrils (and their terminal groups) was constant throughout the process.

Kinetically, a two-stage mechanism appears likely: the rate of defibrillation did not depend on BRC4 concentration, which may be explained by a rapid BRC4 terminal binding, possibly followed by a rate-determining loss of a BRC4-bound protomer unit (Figure 2B, bottom and Figure 2C, bottom). A similar mechanism was previously suggested for the assembly of RAD51 fibrils on DNA assisted by BRCA2 [16,24]. From a thermodynamic point of view, the relatively high value of *k_d_* calculated for the individual steps of RAD51 loss indicates that the driving force of the process may not be a high affinity of the peptide for its fibrillar substrate but rather a “domino” combination based on (i) the coupling between successive RAD51 losses, so that the removal of RAD51 from an *n*-mer shifts to the right with all equilibria involving larger fibrils; and (ii) the thermodynamically favoured complexation of BRC4 to a free protomer, which acts as a final “sink”. The data reported here are, to our knowledge, the first detailed description of the mechanism of disassembly of RAD51 self-assembled fibrils in the absence of DNA.

Our results show that RAD51 self-assembled fibrils were strikingly similar to those previously reported in the presence of DNA [30]. Similarities in the mechanisms of fibrils assembly and disassembly in the absence and presence of DNA were also observed. These data strongly suggest that the intrinsic tendency of the protein to form fibrils, with an intriguing helical shape also in the absence of DNA, is critical to determining the function of the protein regardless of which subcellular compartment it is in. Moreover, the RAD51 mode of interaction with its BRCA2 partner also seems to be preserved in the absence of DNA. The presence of the other cofactors (DNA, ATP, Mg^2+^, etc.) should be pivotal in determining RAD51 conformational stability, availability to heterologous interactions, and in triggering DNA repairing activity intrinsically dependent on the innate protein tendency towards fibrils formation. The detailed mechanistic description of BRC4 binding to RAD51 with dramatic (but close to stoichiometric) morphological consequences is also key to interpreting the biological finding that the peptide inhibits the HR pathway once internalized into the cell. Indeed, BRC4 can bind RAD51, but cannot further support RAD51 activity within the HR pathway as is physiologically performed by BRCA2 [23]. This is because it lacks the BRCA2 NLS. Even upon DNA damage (CPL, Doxo), RAD51 cannot localize at the site of damage in the presence of BRC4.

Small peptides, the stretch of the BRC4 repeat [31,32], and small organic molecules mimicking BRC4 and inhibiting RAD51-BRCA2 interaction have already been reported in the literature [10,33,34,35]. Nevertheless, the BRC4 peptide had a higher affinity compared to nonpeptidic compounds. BRC4 turned out to be the most appropriate tool to shed further light on a key step of the complex HR pathway, in which inhibition, through peptides or small molecules, could be crucial for exploiting innovative anticancer mechanisms.

In our experimental conditions, the peptide (BRC4myr, see Materials and Methods Section 4.1 for further details) prevented RAD51 nuclear localization upon DNA damage, consequently affecting cell proliferation after treatment with DNA-damaging agents. The absence of RAD51 in the nucleus makes cancer cells unable to repair damaged DNA, potentially leading to synthetic lethality. We are now pursuing smart formulations, nanoparticles, and intelligent deliveries to improve BRC4 drug-likeness for its possible exploitation in oncology.

## 4. Materials and Methods

### 4.1. Protocol for the Expression and Purification of His-RAD51

The full-length human RAD51 was expressed in *E. coli* Rosetta2(DE3) pLysS cells carrying a pET15b plasmid. A 6× His tag was inserted at the N terminus of the protein. *E. coli* cells were grown in a TB-5052 auto-induction medium at 20 °C for 72 h. The cell pellet was resuspended in an appropriate volume (10 mL per g of wet pellet) of buffer A (20 mM Tris-HCl (pH 8.00), 500 mM NaCl, 10 mM imidazole, 5 mM DTT, 10% (*v*/*v*) glycerol) supplemented with protease inhibitor cocktail (SIGMAFAST Protease Inhibitor Cocktail Tablets, EDTA. Free). The cell suspension was lysed on ice trough sonication (24 rounds of 30; amplitude 85%; Tip MS72; Bandelin Sonoplus HD2070 sonicator). The disrupted cell suspension was centrifuged for 1 h at 20,000 rpm and 4 °C. All subsequent chromatographic steps were performed at room temperature. The supernatant fraction was applied onto a His-Trap HP (5 mL GE, Healthcare) column equilibrated in buffer A. Bound protein was eluted using a linear gradient from 10% to 100% of buffer B (20 mM Tris-HCl (pH8.00), 500 mM NaCl, 500 mM imidazole, 10% (*v*/*v*) glycerol). Collected fractions corresponding to the recombinant protein were dialyzed overnight at 4 °C against buffer C (50 mM Tris-HCl (pH 8.00), 200 mM KCl, 0.25 mM EDTA, 2 mM DTT, 10% (*v*/*v*) glycerol). Dialyzed protein was loaded onto an anion exchange column (ResQ, GE Healthcare) equilibrated in buffer C. The elution was performed with a linear gradient of buffer D (50 mM Tris-HCl (pH 8.00), 1 M KCl, 0.25 mM EDTA, 2 mM DTT, 10% (*v*/*v*) glycerol). Fractions containing His-RAD51 were pooled and dialyzed against the storage buffer (20 mM Hepes (pH 8.00), 250 mM KCl, 0.1 mM EDTA, 2 mM DTT, 10% (*v*/*v*) glycerol). The protein yield was determined from the optical absorption at 280 nm (extinction coefficient 14,900 M^−1^cm^−1^) of the final sample. The BRC4 peptide and the derivatives used in this work were purchased from Thermo Fisher Scientific. The BRC4 peptide corresponds to the fourth repeat of the BRCA2 sequence (residues 1517–1551) with a molecular weight of 3957.6 Da. The scrambled BRC4 peptide is the BRC4 (35 residues) peptide, carrying a double mutation [F1524A, T1526A] (MW 3851.47 Da). The myrBRC4 peptide was produced by inserting a myristoylated lysine at the C terminal: KEPTLLGFHTASGKKVKIAKESLDKVKNLFDEKEQ[C][C][K(myristoyl]KKK-NH2, (MW 4902.47 Da).

### 4.2. Physico-Chemical Characterization

Size exclusion chromatography (SEC). The recombinant pure human RAD51 protein was incubated at 37 °C for 1 h in the absence or presence of the BRC4 peptide in a 1:4 molar ratio. After incubation, protein samples were loaded onto a size exclusion chromatographic column (Superdex200 Increase 10/300 GL, GE Healthcare, Chicago, IL, USA) equilibrated with the following buffer: 20 mM Hepes (pH 8.00), 250 mM KCl, EDTA 0.1 mM, glycerol 5%, DTT 2 mM.

Static light scattering (SLS) and composition-gradient multi-angle static light scattering (CG-MALS). Measurements were carried out using a Calypso automated delivery system as a mixing unit, connected to a Dawn Heleos II multi-angle light scattering (MALS) and a T-rEX refractive index (RI) detector, both operating at 660 nm (all instruments produced by Wyatt Technology, Santa Barbara, CA, USA). The Calypso software was used to collect and analyze the refractive index and SLS data, assuming dn/dc = 0.185 L/g for both RAD51 and BRC4 and that the actual concentration of all components equated their nominal values. Injection volume and flow rate were fixed at 0.8 mL and 1 mL/min, respectively. A delay time of 180 s was set between each injection. RAD51, BRC4, and its scrambled version were dissolved in a buffer based on 20 mM HEPES adjusted to pH 8 and supplemented with 0.1 mM EDTA, 250 mM KCl, 5% *v/v* glycerol, and 2 mM DTT.

Characterization of RAD51 fibrils. RAD51 solutions were obtained in situ within the Calypso mixing unit by diluting a stock solution with its own buffer and producing RAD51 at five concentrations between 0.1 and 0.35 mg/mL (2.5–8.75 µM). The scattering data were fitted using the Debye formalism (1st order in concentration and 2nd order in angle), yielding weight average mass ($\bar{M_{w}}$), radius of gyration, and second virial coefficient of RAD51 oligomers.

Characterization of BRC4-induced RAD51 defibrillation. RAD51 (constant concentration of 0.25 mg/mL (6.25 µM)) was exposed to either BRC4 or its scrambled version (at final concentrations ranging from 0 to 0.19 mg/mL (48 µM). Light scattering data were used to calculate the apparent weight average mass and radius of gyration.

Dynamic Light Scattering (DLS). A total of 100 μL of the RAD51–peptide mixtures were manually collected at the end of the analysis above and analyzed at a temperature of 25 °C with a Möbiuζ instrument (Wyatt Technology, Santa Barbara, CA, USA), which was equipped with a laser at 532 nm and operated at a scattering angle of 163.5°. DLS acquisition time and number of acquisitions were set at 3 s and 10 acquisitions, respectively, and three measurements were performed on each sample. Correlation functions were analyzed using the Dynals algorithm to yield hydrodynamic size and size polydispersity (PDI) for each sample. Batch measurements were performed on 1.08 mg/mL (2.7 µM) RAD51 solutions in the same buffer in a Zetasizer Nano Particle Analyzer (Malvern, Malvern, United Kingdom) at a temperature of 25 °C.

Microscale Thermophoresis (MST). 6xHis-RAD51 was labelled with the Monolith His-Tag Labeling Kit RED-tris-NTA 2nd Generation kit (NanoTemper Technologies, München, Germany), which specifically recognizes the hexahistidine tail of the recombinant protein. MST measurements were simultaneously performed on 16 capillaries containing a constant concentration (25 nM) of labelled RED-tris-NTA 2nd Generation 6xHis-RAD51 protein and 16 different concentrations of the peptide BRC4. This allowed us to determine a concentration-dependent MST binding curve. The highest BRC4 concentration tested was 32 μM. Binding curves were fitted using the Affinity Analysis software of Nanotemper Technologies to obtain binding affinity data. Binding tests were also performed for the scBRC4 peptide; the highest concentration tested was 400 μM.

Transmission Electron Microscopy (TEM). (A) Negative staining. Recombinant pure 6xHis-RAD51 protein (0.1 mg/mL (2.5 µM)) alone or in the presence of different concentrations of the BRC4 or scBRC4 peptides was incubated for 2 h at 4 °C. After incubation, each sample was adsorbed into pure carbon film 300-mesh copper grids (Electron Microscopy Sciences, Hatfield, PA, USA). After several washes in RAD51 storage buffer (20 mM Hepes (pH 8.00), 250 mM KCl, 0.1 mM EDTA, 2 mM DTT, 10% (*v*/*v*) glycerol), each sample was negatively stained using 1% uranyl acetate in MQ water. The samples were observed with a JEM-1011 (JEOL) transmission electron microscope (TEM) with a thermionic source (W filament) and maximum acceleration voltage of 100 kV equipped with a Gatan Orius SC1000 series CCD camera (4008 × 2672 active pixels), fiberoptically coupled to high-resolution phosphor scintillator. The fibril length and number of RAD51s (alone or in complex with different amounts of BRC4 peptide) were measured using Fiji ImageJ [36]. In total, more than 2500 fibrils from different negative staining experiments were measured for quantification.

(B) Negative staining coupled with streptavidin–gold labelling. Drops of 5 μL of recombinant pure 6xHis-RAD51 protein (0.1 mg/mL (2.5 µM)) alone or in the presence of different concentrations of BRC4 were incubated for 30–90 s on plasma-cleaned carbon-film-coated 300-mesh nickel grids (Electron Microscopy Sciences, Hatfield, PA, USA). After two washing steps with drops of 50 μL of washing buffer (0.1% bovine serum albumin (BSA) (Miltenyi Biotec, Bergish, Germany) in phosphate-buffered saline (PBS) (Sigma-Aldrich, St. Louis, MO, USA), grids were incubated for 30 min (at room temperature) or 2 h (at 4 °C) with streptavidin–gold (Sigma-Aldrich, St. Louis, MO, USA) diluted at 1:20–1:60 in washing buffer. The grids were then washed with several drops of RAD51 storage buffer, negatively stained, and imaged as described (see above). The streptavidin–gold labelling quantification was performed manually or automatically using the Fiji ImageJ software. We quantified a total area of about 220 µm^2^, corresponding to more than 90 micrographs (40 micrographs for RAD51-BRC4 complex (CTR) and 50 micrographs for RAD51-biotinilated BRC4 complex) from several experiments. Statistical analysis was performed with OriginPro 9.1.0.

### 4.3. Biological Characterization

General cell culture. BxPC-3 cells (purchased from ATCC) were grown in RPMI 1640 containing 10% FBS, 100 U/mL penicillin–streptomycin, and 2 mM glutamine. All media and supplements were from Sigma-Aldrich. Cultures were routinely tested for mycoplasma contamination.

Homologous Recombination assay. Homologous recombination (HR) was assessed using a commercially available assay (Norgen, Thorold, ON, Canada). This assay is based on cell transfection with two plasmids that recombine upon cell entry. The efficiency of HR was assessed by real-time PCR, using primer mixtures included in the assay kit. Different primer mixtures allow differentiation between the original plasmid backbones and their recombination product. Briefly, BxPC3 cells (2 × 10^5^ per well) were seeded in a 24-well plate and allowed to adhere overnight. Cotransfection with the two plasmids was performed in Lipofectamine 2000 (Invitrogen, Waltham, MA, USA), according to the manufacturer’s instructions. During 5 h of transfection, cells were exposed to different doses of peptides, dissolved in PBS. After washing with PBS, cells were harvested, and DNA was isolated using QIAamp DNA Mini kit (Qiagen, Hilden, Germany). Sample concentration was measured using an ONDA Nano Genius photometer. The efficiency of HR was assessed by real-time PCR, using 25 ng of template, primer mixtures included in the assay kit, and protocol indicated by the manufacturer. Data analysis was based on the ΔΔCt method: (Recombination Product/Backbone Plasmids) treated versus (Recombination Product/Backbone Plasmids) control.

Immunofluorescence. Immunofluorescence was used to study RAD51 nuclear translocation. To visualize RAD51 in cell nuclei, BxPC-3 cells were seeded on glass coverslips placed in a 6-well culture plate (2 × 10^5^ cells/well) and allowed to adhere for 24 h. Cultures were pre-incubated in RPMI without serum with 5 µM BRC4myr or scBRC4myr for 1 h and subsequently exposed to 50 μM cisplatin (MedChemExpress) or 200 nM doxorubicin (Selleckchem) for 1.5 h. The medium was then removed to eliminate the chemotherapeutic agents, and cells were maintained in the presence of 5 µM BRC4myr for an additional 4 h. After this time, cells were fixed with PBS containing PFA 4% for 15 min, permeabilized in 70% ethanol, and then washed twice in PBS. The samples were incubated in 10% bovine serum albumin (BSA) (Sigma-Aldrich) in PBS for 30 min at 37 °C and subsequently in the primary rabbit polyclonal antibody anti-RAD51 (BioAcademia 1:1000 in 5% PBS/BSA) overnight at 4 °C. After washing, coverslips were incubated in secondary anti-rabbit rhodamine-labelled antibody (Novus Biologicals, 1:1000 in 1% BSA/PBS) for 30 min at 37 °C, washed, air-dried, and mounted in a solution 2 µg/mL DAPI in DABCO. Images were acquired using a Nikon fluorescent microscope equipped with filters for TRITC and DAPI. The percentage of cells bearing nuclear foci was estimated by analyzing approximately 300 cells for each treatment sample.

Cell viability. Cell viability was assessed with the CellTiter-Glo Luminescent Cell Viability Assay from Promega. For this experiment, 2 × 10^4^ cells were seeded using complete medium in a 96-multiwell white body plate and allowed to adhere overnight. The next day, the medium was replaced, and treatments were conducted entirely in the absence of serum. After a brief wash, cells were pre-incubated with 5 µM BRC4myr for 1 h. Where indicated, cisplatin (50 and 100 μM) or doxorubicin (200 nM and 1 μM) was subsequently added to wells for 1.5 h. The medium was then removed to eliminate the chemotherapeutic agents, and cells were maintained in the presence of 5 µM BRC4myr for an additional 48 h. After treatments, the multiwell plate was allowed to equilibrate at room temperature for 30 min, and the CellTiter-Glo reactive was directly added to each well. The plate was kept on a shaker for 10 min to induce cell lysis, and its luminescence was measured following manufacturer’s instructions using a Fluoroskan Ascent FL reader (Labsystems, Vantaa, Finland).

## Supplementary Materials

The following supporting information can be downloaded at: https://www.mdpi.com/article/10.3390/ijms23158338/s1. Reference [37] is cited in the supplementary materials.

## Appendix A

**Defibrillation model**

Apparent weight average mass data ($\bar{AM_{w}}$) were obtained from SLS analysis. The data indicate the mass averaged through the various species in the sample, i.e., fibrils (with and without bound peptides), free peptides, and peptide–protein complexes.

(A1) $$\bar{AM_{w}}=W_{BRC4}\cdot MW_{BRC4}+W_{RAD51-BRC4}\cdot MW_{RAD51-BRC4}+\underset{i}{\sum }{\left(W_{Fibril}\right)}_{i}\cdot {\left(M_{Fibril}\right)}_{i}$$

where W, MW, and $M_{Fibril}$ are the mass fraction of each species, the molecular weight of each molecular species (the free peptide and the RAD51-BRC4 complex), and the mass of a given fibril, respectively. In the sample, the mass fraction of a species *i* is a function of its mass $M_{i}$ (a molecular weight or a fibril mass), its molar concentration, and the total concentration of the colloidal species in the sample ($C_{TOT}$_,_ expressed in g/L), which allows Equation (A2) to be transformed into the following expression.

(A2) $$\bar{AM_{w}}=\frac{1}{C_{TOT}}\cdot ({\left[BRC4\right]}_{eq}\cdot MW_{BRC4}^{2}+\left[RAD51-BRC4{]}_{eq}\cdot MW_{RAD51-BRC4}^{2}+\underset{i}{\sum }{\left[Fib_{i}\right]}_{eq}\cdot {\left(M_{Fibril}\right)}_{i}^{2}\right)$$

We then took a mathematical approach that is conceptually similar to those often used in step-growth (equilibrium) polymerization. This approach is based on the estimation of dissociation constants through kinetic equations, allowing the system to reach the steady state at each defibrillation step. To do so in a computationally efficient (although very approximated) way, we introduced the following assumptions:

(i) The defibrillation processes comprise consecutive steps where a single protomeric unit is removed, and each step is characterized by the same dissociation constant *k_d_* (implying that the loss of a unit is equally likely in a longer or in a shorter fibril);

(ii) In the absence of BRC4, all fibrils have an identical length equal to 71 protomers. Thus, before the addition of the peptide, the sum in Equations (A2) and (A3) is reduced to a single value with *I* = 71. Clearly, it is unrealistic to ignore the original width of the fibril size distribution. However, this assumption allows for a much clearer model of defibrillation.

Importantly, *I* = 1 corresponds to the particular situation of a “monomeric” fibril, i.e., the uncomplexed RAD51 unit. In this case, the equilibrium to be considered is analogous to that of defibrillation steps, but the complexation of BRC4 with a fibril yields two products (i.e., RAD51-BRC4 and a shorter fibril), while the complexation of BRC4 with RAD51 yields only one product (i.e., RAD51-BRC4). This means that the corresponding dissociation constants are dimensionally different: the *k_d_* for the former is dimensionless; the *k_d1_* for the latter has the units of a concentration.

In the model, we have considered that in each step of the process, the rate of loss and the rate of addition of protomers on a given fibril must be identical at equilibrium. The time evolution of the process in each defibrillation step can be described by a system of coupled ordinary differential equations corresponding to second-order reactions, such as those shown in Equation (A3):

(A3) $$\{\begin{array}{c} \frac{d[Fib_{i}]}{d\tau }=-[Fib_{i+1}]\left(\tau \right)\cdot \frac{\left[BRC4\right]\left(\tau \right)}{{\left[RAD51\right]}_{0}}+k_{d}\cdot [Fib_{i}]\left(\tau \right)\cdot \frac{{\left[BRC4\right]}_{0}-\left[BRC4\right]\left(\tau \right)}{{\left[RAD51\right]}_{0}}\\ \frac{d}{d\tau }[Fib_{1}]=-[Fib_{1}]\left(\tau \right)\cdot \frac{\left[BRC4\right]\left(\tau \right)}{{\left[RAD51\right]}_{0}}+k_{d1}\cdot \frac{{\left[BRC4\right]}_{0}-\left[BRC4\right]\left(\tau \right)}{{\left[RAD51\right]}_{0}} \end{array}\;$$

In Equation (A3) we used τ as an arbitrary scaled, dimensionless time, which is formally obtained by dividing time by the forward kinetic constant and by ${\left[RAD51\right]}_{0}$. Under the already described assumption of fibril monodispersity (${\left[Fib\right]}_{i}\left(\tau =0\right)=\frac{1}{71}\delta_{i,71}$), and using the $\bar{AM_{w}}$ data obtained via SLS as inputs, we iteratively evaluated the time evolution of the system in Equation (A3) independently for the different BRC4/RAD51. After an initial choice of *k_d_* and *k_d_*_1_, the steady-state concentrations for all involved species were calculated, and then both constants were iteratively optimized according to a nonlinear optimization protocol. The cost function minimized by this protocol was the discrepancy between the simulated and observed $\bar{AM_{w}}$ values at these steady states.
