# Supplementary material for: The Mechanistic Understanding of RAD51 Defibrillation: A Critical Step in BRCA2-Mediated DNA Repair by Homologous Recombination
Source: Int J Mol Sci. 2022 Jul 28;23(15):8338. doi: 10.3390/ijms23158338 (PMC9368738; doi:10.3390/ijms23158338)
Supplement: Supplementary file 1 [file ijms-23-08338-s001.zip › ijms-1798364-supplementary.pdf]

SUPPORTING INFORMATION

The mechanistic understanding of RAD51 defibrillation: a critical step in BRCA2-mediated DNA repair by homologous recombination

Fabrizio Schipani[1], Marcella Manerba[1], Roberto Marotta[2], Laura Poppi[3] Arianna Gennari[4], Francesco Rinaldi[1], Andrea Armirotti[5], Fulvia Farabegoli[3], Marinella Roberti[3], Giuseppina Di Stefano[6], Walter Rocchia[7], Stefania Girotto[1,], Nicola Tirelli[4,8,], Andrea Cavalli[1,3,*]*

[1]Computational and Chemical Biology, Istituto Italiano di Tecnologia, Via Morego 30, 16163 Genoa, Italy.

[2]Electron Microscopy Facility, Istituto Italiano di Tecnologia, via Morego, 30, 16163 Genoa, Italy.

[3]Department of Pharmacy and Biotechnology, University of Bologna, Via Belmeloro 6, 40126 Bologna, Italy

[4]Laboratory of Polymers and Biomaterials, Istituto Italiano di Tecnologia, via Morego, 30, 16163 Genoa, Italy.

[5]Analytical Chemistry Lab, Istituto Italiano di Tecnologia, via Morego, 30, 16163 Genoa, Italy.

[6]Department of Experimental, Diagnostic and Specialty Medicine, University of Bologna, Via S. Giacomo 14, 40126 Bologna, Italy.

[7]CONCEPT Lab, Istituto Italiano di Tecnologia, via E. Melen 83, 16152 Genoa, Italy.

[8]School of Health Sciences, University of Manchester, Oxford Road, Manchester M13 9PL, United Kingdom.

*Andrea Cavalli, Computational and Chemical Biology, Istituto Italiano di Tecnologia, Via Morego 30, 16163 Genoa, Italy, phone: +39 0102897403; Nicola Tirelli, Laboratory of Polymers and Biomaterials, Istituto Italiano di Tecnologia, via Morego, 30, 16163 Genoa, Italy, phone: +39 0102896923.

Email: andrea.cavalli@iit.it, nicola.tirelli@iit.it

1SI. Physico-chemical behaviour of RAD51, alone and with BRC4

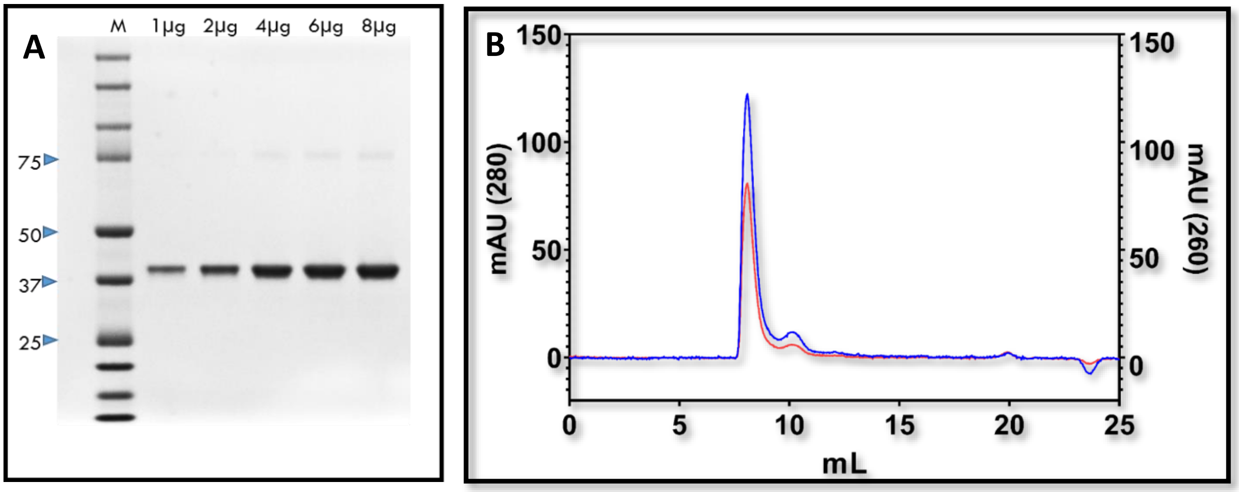

Figure S1. (A) SDS-PAGE analysis of increasing quantities of purified recombinant full-length RAD51.
(B) Size exclusion chromatographic (Superdex200 Increase 10/300 GL, GE Healthcare) elution profile of RAD51 recorded at 280 nm absorbance (blue line, left axis) and at 260 nm absorbance (red line, right axis).Data suggest that the recombinant RAD51 is pure and no DNA contamination is present.

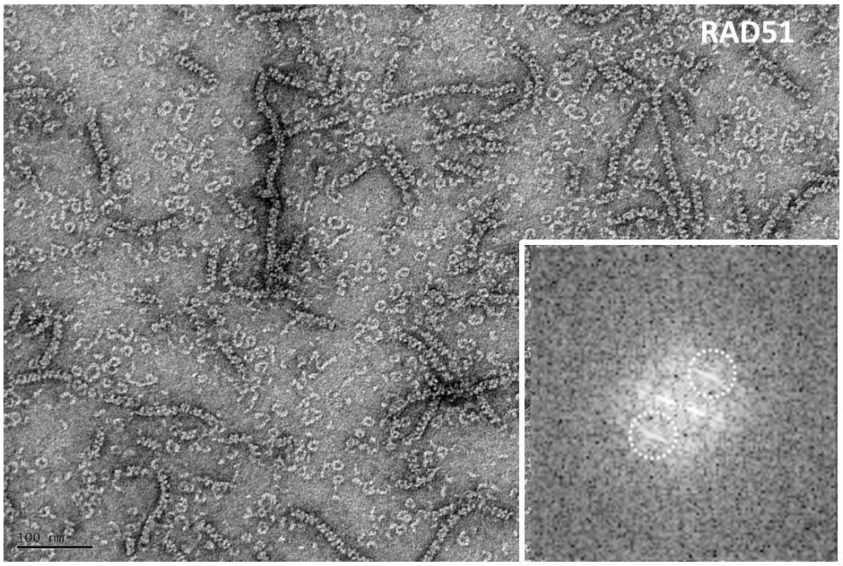

Figure S2 : Negative staining TEM of RAD51 (0.1 mg/ml (2.5 μM)) fibrils. Inset: fibril Fast Fourier Transform (FFT) (spot @6.5 nm are circled). The FFT shows smeared FFT maxima similar to layer lines pointing out a fibril helical organization.

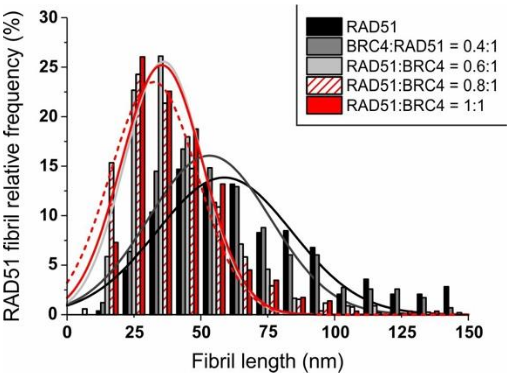

Figure S3. RAD51 fibril length number distributions as a function of RAD51/BRC4 molar ratio. The curves show the results of fittings with a Gaussian model.

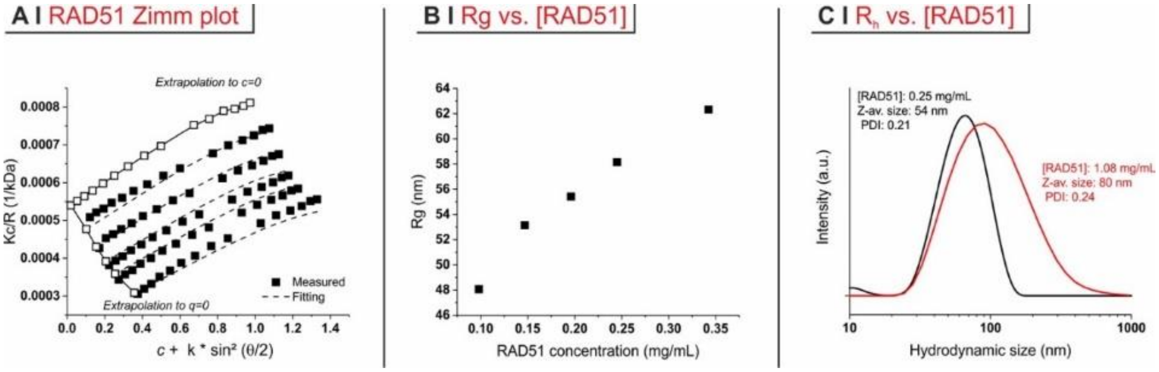

Figure S4. ***A***. Zimm plot for RAD51 in a modified HEPES buffer at pH 8; q is the scattering vector (=4π/λ*sin(θ/2)). ***B***. Rg values derived from the angular dependency of scattering intensity are reported as a function of RAD51 concentration. ***C***. DLS intensity size distributions for RAD51 at 0.25 mg/mL (black) and 1.08 mg/mL (red).

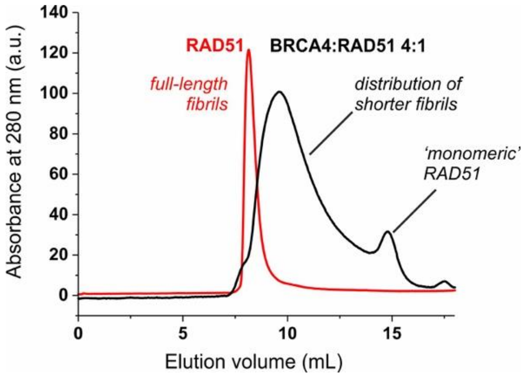

Figure S5. Size exclusion chromatography (SEC) analysis of RAD51 before (red line) and after (black line) incubation with BRC4 (1 h, 37 °C; RAD51:BRC4 = 1:4; see Materials and Methods, section 4.2 for SEC conditions). Please note that the 'monomeric' peak of RAD51 is caused by this form being the terminal step of the defibrillation process.

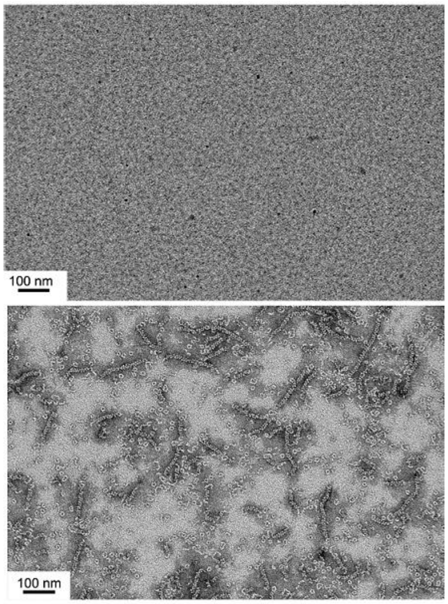

Figure S6. Negative staining TEM of 0.1 mg/mL RAD51 fibrils incubated for 2 h with BRC4 peptide (RAD51/BRC4 1:4 molar ratio) (upper panel) or scBRC4 peptide (RAD51/scBRC4 1:4 molar ratio) (lower panel) clearly shows that the double mutated peptide does not affect, or marginally affects, RAD51 fibrils, while the BRC4 peptide, under the same conditions, induces a substantial disassembly of RAD51 fibrils.

Table S1. Structural features of RAD51 oligomers extrapolated at infinite dilution and at 0.25 mg/mL (6.25 μM).

**RAD51 conc.**	**Weight-aver. mass** (kDa)	**Aggr. num.**	**Rg** (nm)	**Rg / Rh**	**A2** (mmol*mL/ g^2)	**Num.-aver. fibril length** (nm)	**Weight-aver. fibril length** (nm)
Inf. dil.	1634 ± 25 [a]	≈40 [c]	49 ± 1[a]		-878 ± 5 [a]		
0.25 mg/mL	2833 ± 25 [b]	≈70 [c]	59 ± 1	≈2		78 [e]	101[e] / 170 [f]

[a] Values obtained via extrapolation to zero concentration in a Zimm plot comprising RAD51concentrations between 0.1 and 0.35 mg/mL (2.5 - 8.75 μM) (see Figure S4A). By extending the study to lower RAD51 concentrations, the Zimm plot appeared to provide further lower fibrillar masses, but with an unacceptably high noise due to the very low scattering intensity.

[b] This value is obtained from the angular dependency of the scattered intensity at a finite concentration, and therefore is likely to significantly overestimate the real mass of the oligomers.

[c] The aggregation number (= average number of RAD51 units in a fibril) is obtained by dividing the weight-average mass of the oligomers by the molar mass of RAD51 (40 kDa).

[d] The Rg/Rh ratio (aspect ratio) is calculated using the Rg (SLS) and Rh (DLS) values obtained at a RAD51 concentration of 0.25 mg/mL (6.25 μM).

[e] Number- or weight-average fibril length calculated from TEM images.

[f] Weight-average fibril length calculated by assuming a rod geometry for which $\langle {R_g}^2 \rangle = {L^2}/{12} + {a^2}/{2}$ where L is the length and a the radius.[1] The average radius was assumed to be 7.5 nm as from TEM images and Rg is the value measured at 0.1 mg/mL (2.5 μM), i.e. the RAD51 concentration used to prepare the TEM samples.

Reference

1. Wyatt, P. J., Measurement of Special Nanoparticle Structures by Light Scattering. *Anal Chem* **2014,** 86, (15), 7171-7183.
